# Supplementary material for: Antimicrobial Activity of Divaricatic Acid Isolated from the Lichen Evernia mesomorpha against Methicillin-Resistant Staphylococcus aureus
Source: Molecules. 2018 Nov 23;23(12):3068. doi: 10.3390/molecules23123068 (PMC6320781; doi:10.3390/molecules23123068)
Supplement: Supplementary file 1 [file molecules-23-03068-s001.pdf]

Supplementary Material

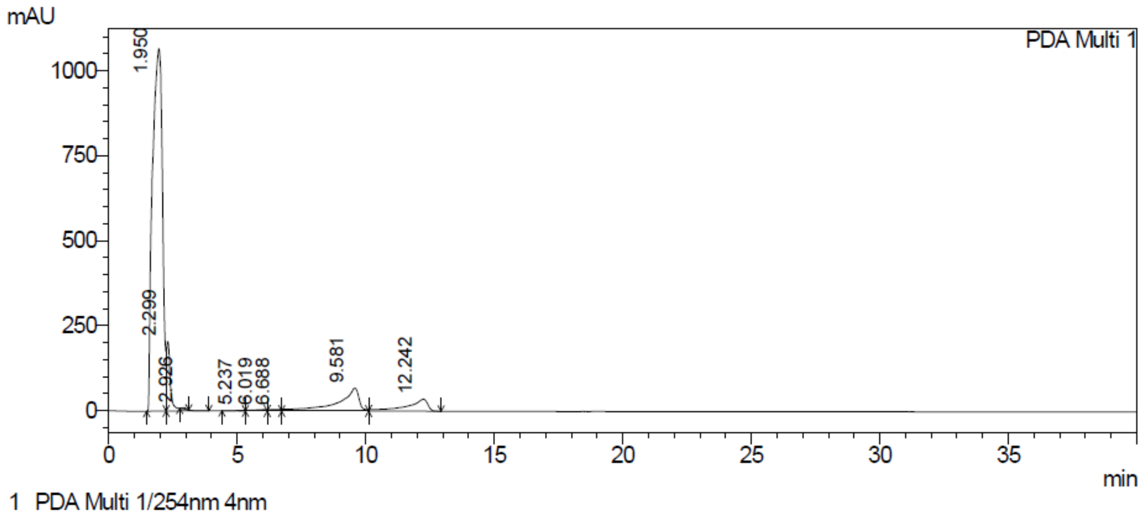

PeakTable

PDA Ch1 254nm 4nm

Peak#	Ret. Time	Name	Area	Area %
1	1.950	Acetone	28723023	77.607
2	2.299	Galbinic acid	2215095	5.985
3	2.926	RT:2.926	40588	0.110
4	5.237	RT:5.237	38971	0.105
5	6.019	RT:6.019	134590	0.364
6	6.688	RT:6.688	126307	0.341
7	9.581	Atranorin	3784272	10.225
8	12.242	Chloroatranorin	1947993	5.263
Total			37010838	100.000

Figure S1. HPLC chromatogram and peak details in sample 412 (*Everniastrum* sp.).

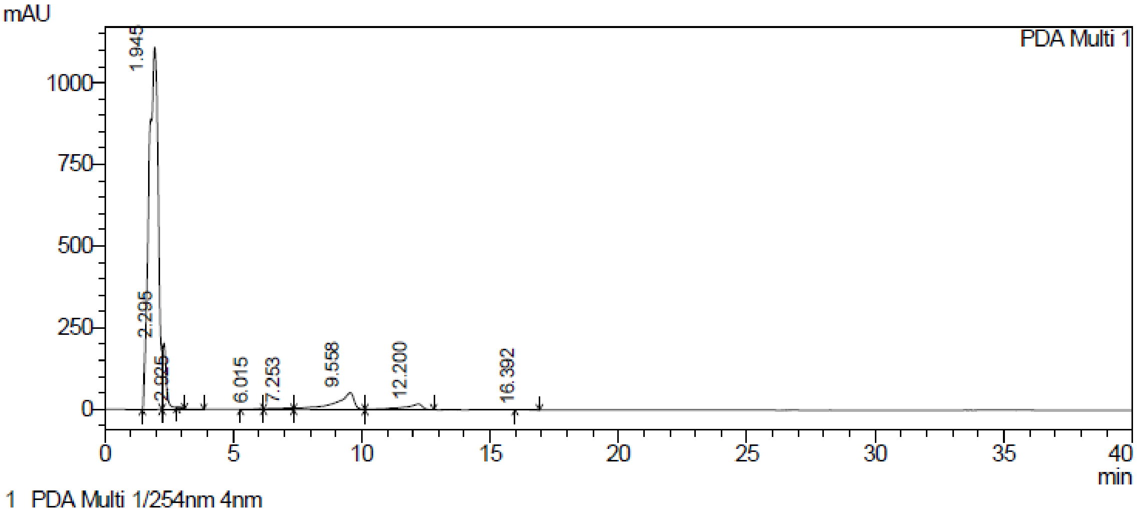

PeakTable

PDA Ch1 254nm 4nm

Peak#	Ret. Time	Name	Area	Area %
1	1.945	Acetone	28474276	82.394
2	2.295	Galbinic acid	2163259	6.260
3	2.925	RT:2.925	24111	0.070
4	6.015	RT:6.015	73050	0.211
5	7.253	RT:7.253	205237	0.594
6	9.558	Atranorin	2660435	7.698
7	12.200	Chloroatranorin	939863	2.720
8	16.392	RT:16.392	18514	0.054
Total			34558744	100.000

Figure S2. HPLC chromatogram and peak details in sample 419 (*Everniastrum* sp.).

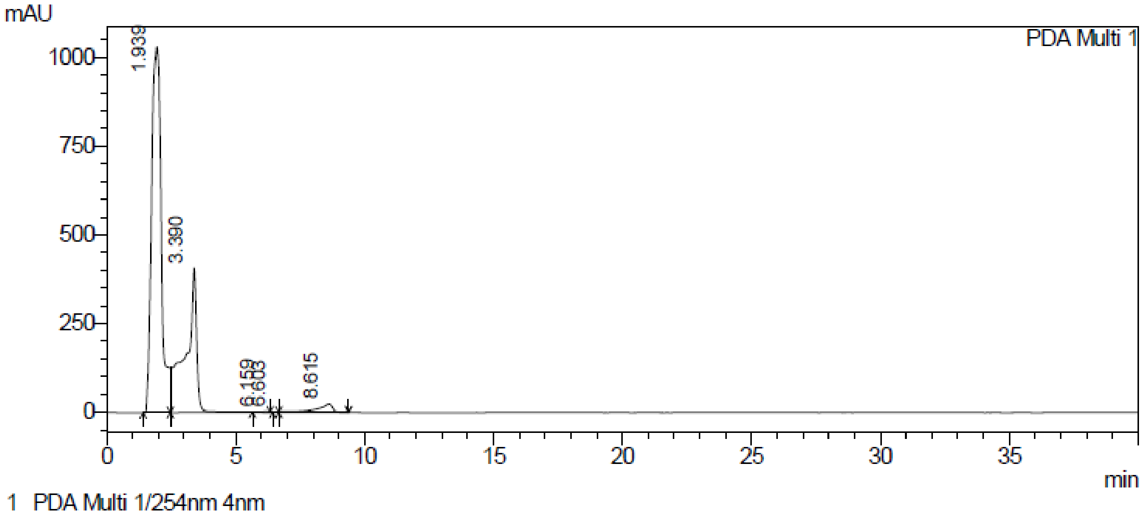

PeakTable

PDA Ch1 254nm 4nm

Peak#	Ret. Time	Name	Area	Area %
1	1.939	acetone	26671375	66.376
2	3.390	Pgrex-1	12549960	31.233
3	6.159	RT:6.159	20881	0.052
4	6.603	RT:6.603	10299	0.026
5	8.615	usnic acid	929638	2.314
Total			40182153	100.000

Figure S3. HPLC chromatogram and peak details in sample 421.

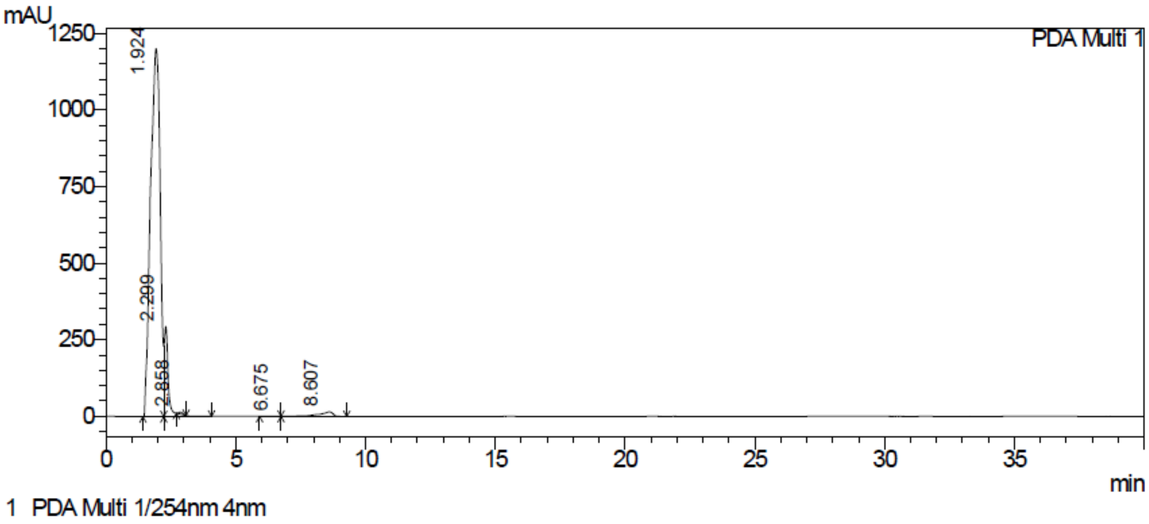

PeakTable

PDA Ch1 254nm 4nm

Peak#	Ret. Time	Name	Area	Area %
1	1.924	acetone	31124214	88.773
2	2.299	galbinic acid	3208325	9.151
3	2.858	RT:2.858	71932	0.205
4	6.675	RT:6.675	24384	0.070
5	8.607	usnic acid	631648	1.802
Total			35060503	100.000

Figure S4. HPLC chromatogram and peak details in sample 422.

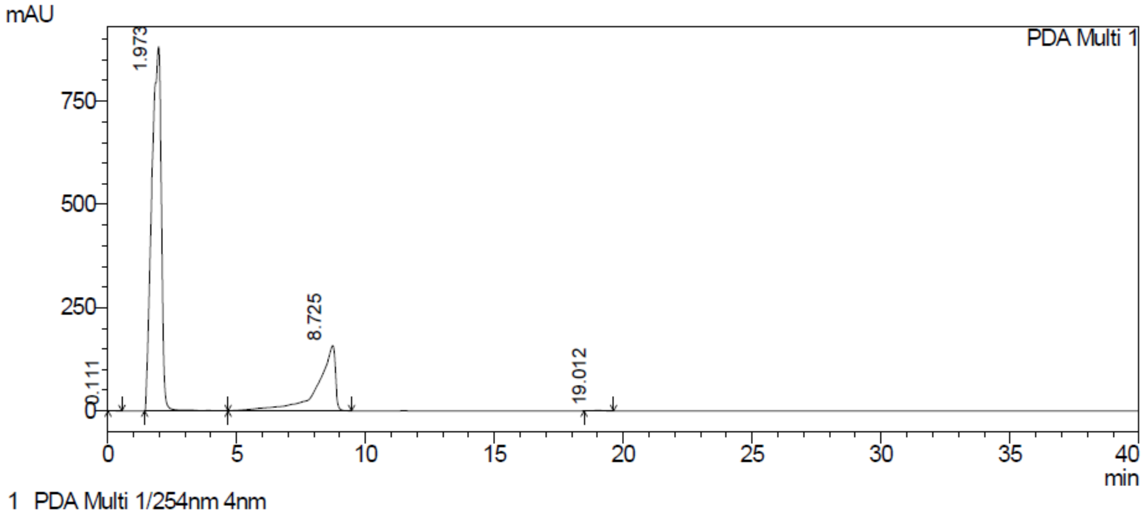

PeakTable

PDA Ch1 254nm 4nm

Peak#	Ret. Time	Name	Area	Area %
1	0.111	RT:0.111	15504	0.051
2	1.973	Acetone	22890873	74.989
3	8.725	Usnic acid	7572417	24.807
4	19.012	RT:19.012	47025	0.154
Total			30525818	100.000

Figure S5. HPLC chromatogram and peak details in sample 435 (*Allocetraria ambigua*).

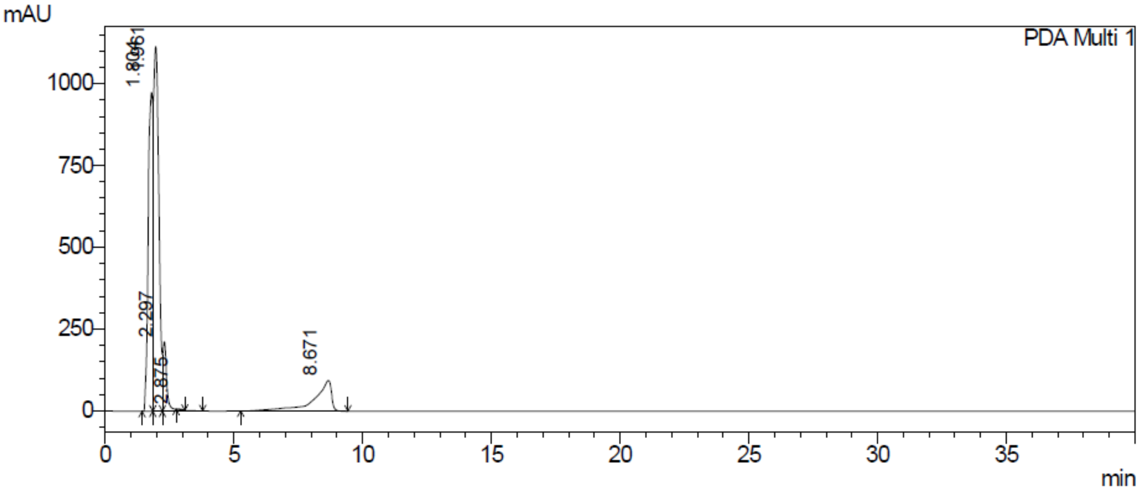

1 PDA Multi 1/254nm 4nm

PeakTable

PDA Ch1 254nm 4nm

Peak#	Ret. Time	Name	Area	Area %
1	1.804	acetone	11698437	33.618
2	1.961	RT:1.961	16493037	47.397
3	2.297	galbinic acid	2282794	6.560
4	2.875	RT:2.875	21810	0.063
5	8.671	usnic acid	4301604	12.362
Total			34797681	100.000

Figure S6. HPLC chromatogram and peak details in sample 442 (*Everniastrum nepalense*).

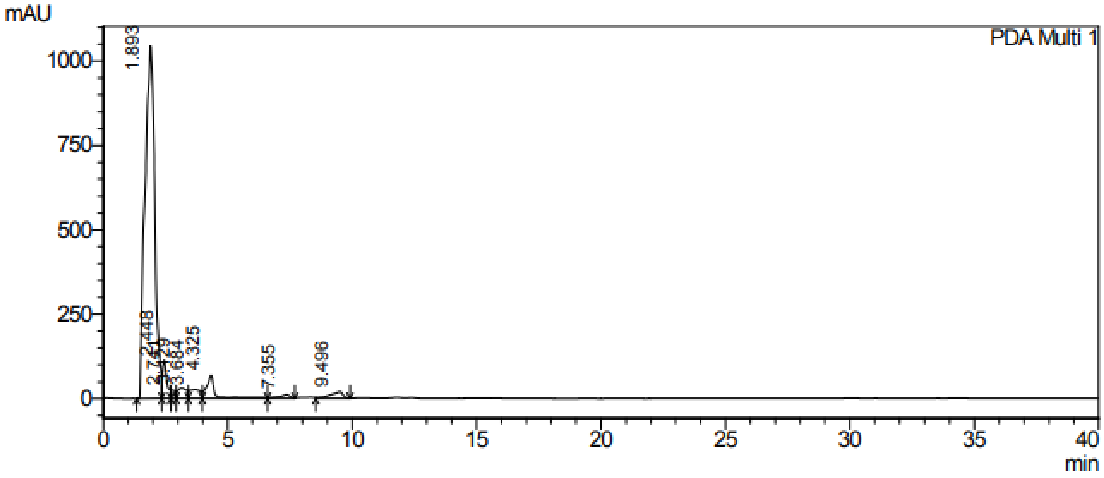

1 PDA Multi 1/254nm 4nm

PeakTable

PDA Ch1 254nm 4nm

Peak#	Ret. Time	Name	Area	Area %
1	1.893	acetone	28708353	82.581
2	2.448	RT:2.448	1397259	4.019
3	2.741	2-0-methylrhizonic acid	316052	0.909
4	3.129	RT:3.129	782480	2.251
5	3.684	RT:3.684	802475	2.308
6	4.325	RT:4.325	1674453	4.817
7	7.355	RT:7.355	373677	1.075
8	9.496	usnic acid	709098	2.040
Total			34763846	100.000

Figure S7. HPLC chromatogram and peak details in sample 473 (*Niebla ceruchoides*).

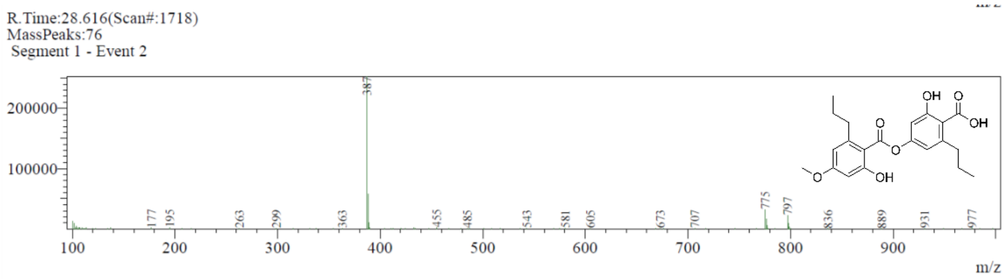

Figure S8. Negative ESI-mass spectrum and chemical structure of divaricatic acid.

$[M-H]^{-} = 387$, real mass $[M] = 388$.

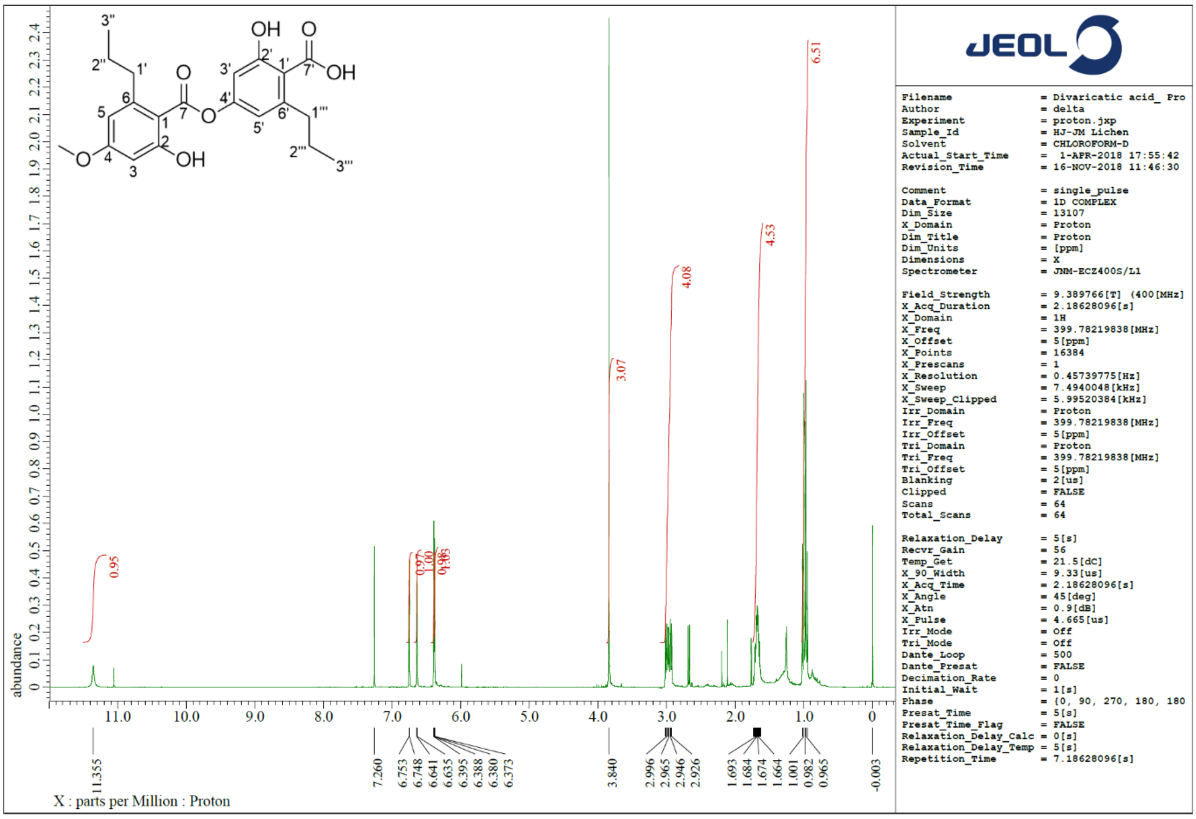

Figure S9. ^{1}H-NMR spectrum of divaricatic acid.

^{1}H-NMR (400 MHz, CHLOROFORM-D) δ 11.35 (s, 1H, COOH), 6.75 (d, $J = 2.1$ Hz, 1H, H-5'), 6.64 (d, $J = 2.3$ Hz, 1H, H-3'), 6.39 (d, $J = 2.7$ Hz, 1H, H-3, 6.38 (d, $J = 2.7$ Hz, 1H, H-5), 3.84 (s, 3H, OCH3), 2.93-3.02 (m, 4H, H-1'', H-1'''), 1.63-1.73 (m, 4H, 2xCH2, H-2'', H-2'''), 0.95(t, J=7.2Hz, 3H, H-3''), 0.98(t, J=7.2Hz, 3H, H-3''')

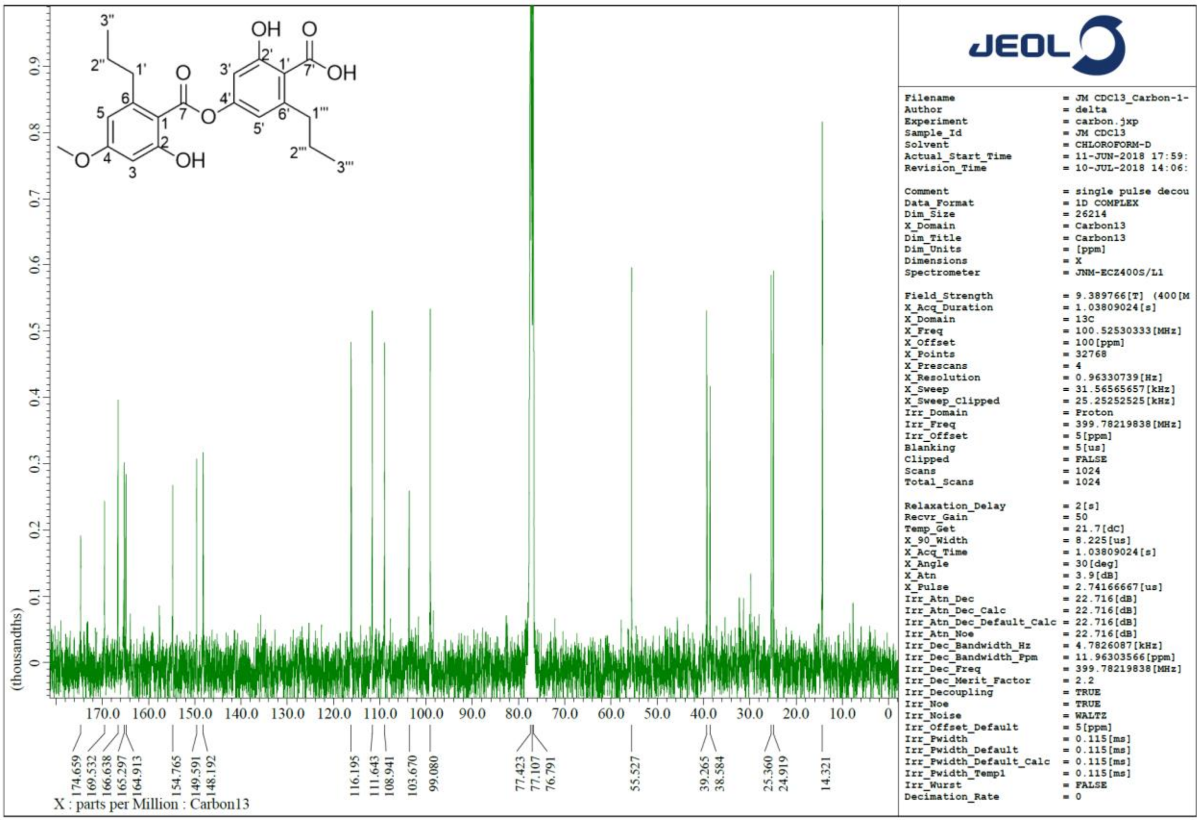

Figure S10. ^{13}C-NMR spectrum of divaricatic acid.

^{13}C-NMR (101 MHz, CHLOROFORM-D) δ 174.95(C-7’), 169.40(C-7), 166.54(C-4), 165.24(C-2), 164.83(C-2’), 154.83(C-4’), 149.62(C-6), 148.08(C-6’), 116.20(C-5’), 111.56(C-3’), 108.91(C-5), 108.69(C-1’), 103.53(C-1), 98.98(C-3), 55.42(OCH3), 39.16(C-1’’’), 38.50(C-1’’), 25.25(C-2’’’), 24.80(C-2’’), 14.21(C-3’’’), 14.19(C-3’’)

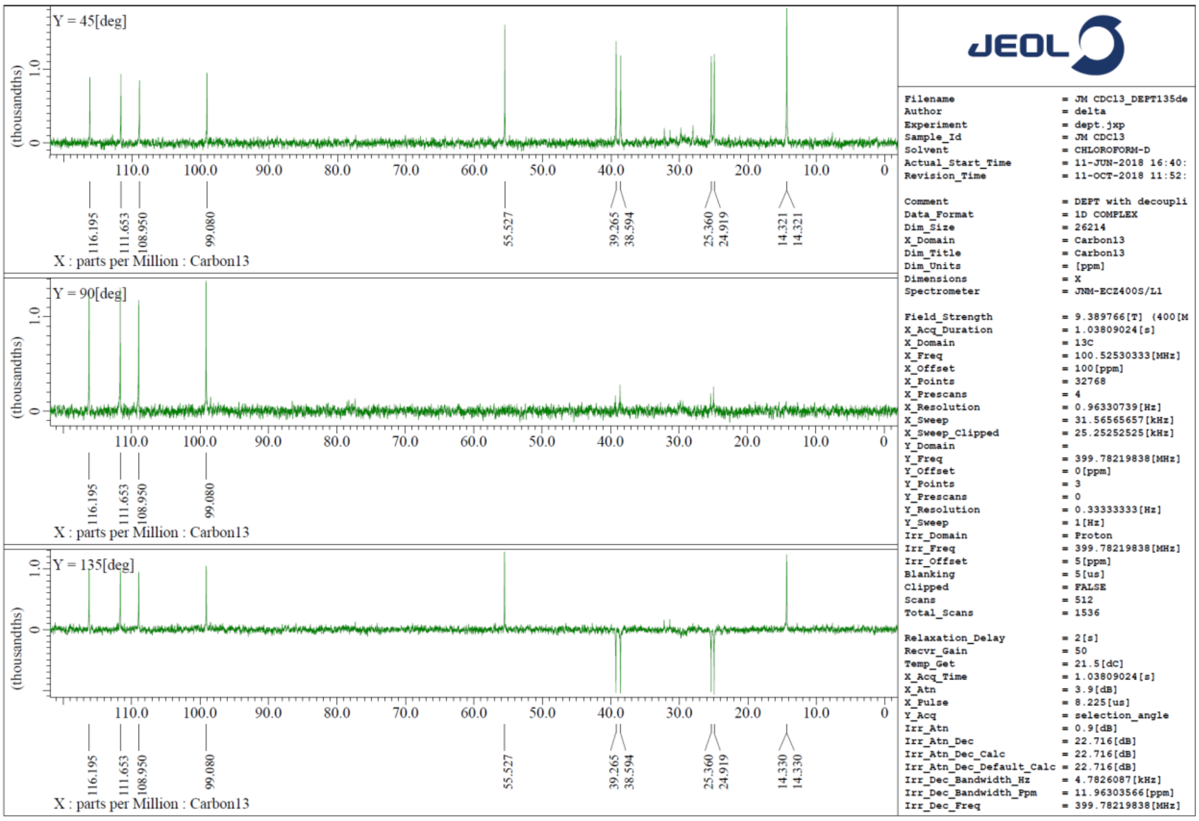

Figure S11. DEPT spectrum of divaricatic acid.

(A)

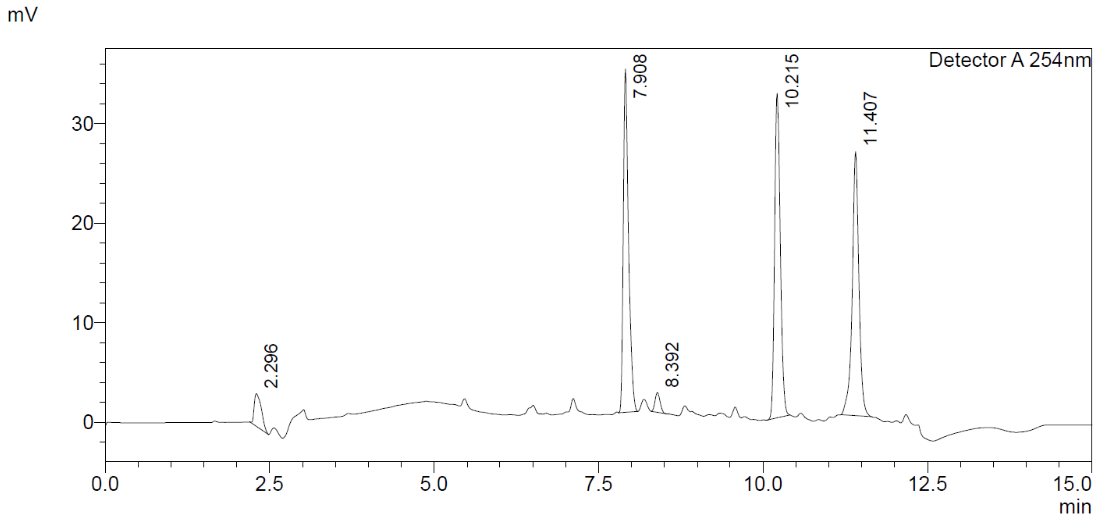

Detector A 254nm

Peak#	Ret. Time	Area	Height	Conc.	Unit	Mark	Name
1	2.296	24916	3270	4.114			
2	7.908	190929	34500	31.527			
3	8.392	10587	1992	1.748			
4	10.215	190239	32630	31.413			
5	11.407	188939	26545	31.198			
Total		605610	98938				

(B)

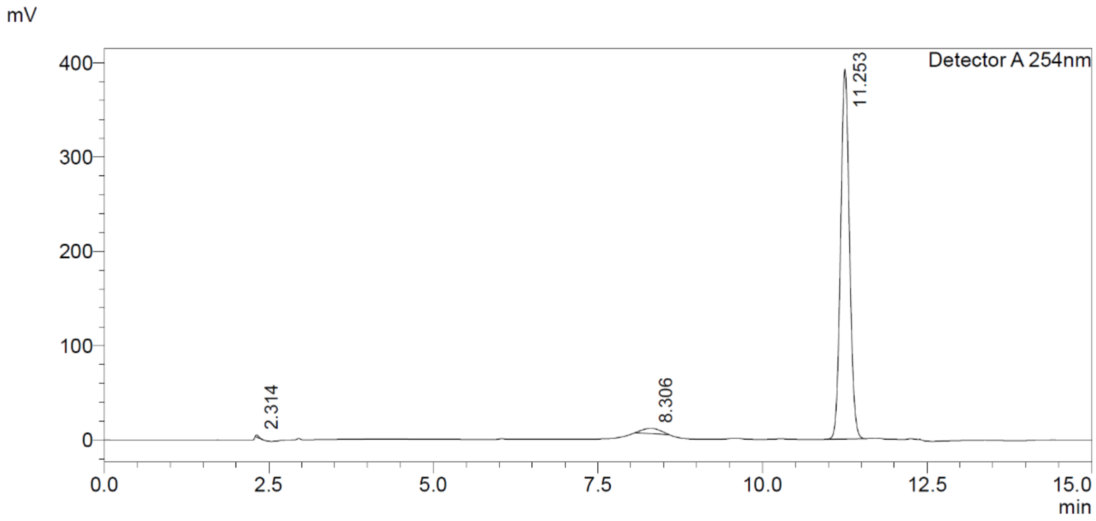

Detector A 254nm

Peak#	Ret. Time	Area	Height	Conc.	Unit	Mark	Name
1	2.314	6206	2383	0.163		M	
2	8.306	105181	5541	2.756		M	
3	11.253	3705398	392128	97.082			
Total		3816785	400052				

Figure S12. LC chromatograms of sample 458 (*Evernia mesomorpha*) (**A**) and purified divaricatic acid (**B**).
